# Systematic Literature Review of E-Learning Capabilities to Enhance Organizational Learning

**DOI:** 10.1007/s10796-020-10097-2

**Published:** 2021-02-01

**Authors:** Michail N. Giannakos, Patrick Mikalef, Ilias O. Pappas

**Affiliations:** 1 Norwegian University of Science and Technology, Trondheim, Norway; 2 University of Agder, Kristiansand, Norway

**Keywords:** Organizational learning, E-learning, Literature review, Learning environments

## Abstract

E-learning systems are receiving ever increasing attention in academia, business and public administration. Major crises, like the pandemic, highlight the tremendous importance of the appropriate development of e-learning systems and its adoption and processes in organizations. Managers and employees who need efficient forms of training and learning flow within organizations do not have to gather in one place at the same time or to travel far away to attend courses. Contemporary affordances of e-learning systems allow users to perform different jobs or tasks for training courses according to their own scheduling, as well as to collaborate and share knowledge and experiences that result in rich learning flows within organizations. The purpose of this article is to provide a systematic review of empirical studies at the intersection of e-learning and organizational learning in order to summarize the current findings and guide future research. Forty-seven peer-reviewed articles were collected from a systematic literature search and analyzed based on a categorization of their main elements. This survey identifies five major directions of the research on the confluence of e-learning and organizational learning during the last decade. Future research should leverage big data produced from the platforms and investigate how the incorporation of advanced learning technologies (e.g., learning analytics, personalized learning) can help increase organizational value.

## Introduction

E-learning covers the integration of information and communication technology (ICT) in environments with the main goal of fostering learning (Rosenberg and Foshay 2002). The term “e-learning” is often used as an umbrella term to portray several modes of digital learning environments (e.g., online, virtual learning environments, social learning technologies). Digitalization seems to challenge numerous business models in organizations and raises important questions about the meaning and practice of learning and development (Dignen and Burmeister 2020). Among other things, the digitalization of resources and processes enables flexible ways to foster learning across an organization’s different sections and personnel.

Learning has long been associated with formal or informal education and training. However organizational learning is much more than that. It can be defined as “a learning process within organizations that involves the interaction of individual and collective (group, organizational, and inter-organizational) levels of analysis and leads to achieving organizations’ goals” (Popova-Nowak and Cseh 2015) with a focus on the flow of knowledge across the different organizational levels (Oh 2019). Flow of knowledge or learning flow is the way in which new knowledge flows from the individual to the organizational level (i.e., feed forward) and vice versa (i.e., feedback) (Crossan et al. 1999; March 1991). Learning flow and the respective processes constitute the cornerstone of an organization’s learning activities (e.g., from physical training meetings to digital learning resources), they are directly connected to the psycho-social experiences of an organization’s members, and they eventually lead to organizational change (Crossan et al. 2011). The overall organizational learning is extremely important in an organization because it is associated with the process of creating value from an organizations’ intangible assets. Moreover, it combines notions from several different domains, such as organizational behavior, human resource management, artificial intelligence, and information technology (El Kadiri et al. 2016).

A growing body of literature lies at the intersection of e-learning and organizational learning. However, there is limited work on the qualities of e-learning and the potential of its qualities to enhance organizational learning (Popova-Nowak and Cseh 2015). Blockages and disruptions in the internal flow of knowledge is a major reason why organizational change initiatives often fail to produce their intended results (Dee and Leisyte 2017). In recent years, several models of organizational learning have been published (Berends and Lammers 2010; Oh 2019). However, detailed empirical studies indicate that learning does not always proceed smoothly in organizations; rather, the learning meets interruptions and breakdowns (Engeström et al. 2007).

Discontinuities and disruptions are common phenomena in organizational learning (Berends and Lammers 2010), and they stem from various causes. For example, organizational members’ low self-esteem, unsupportive technology and instructors (Garavan et al. 2019), and even crises like the Covid-19 pandemic can result in demotivated learners and overall unwanted consequences for their learning (Broadbent 2017). In a recent conceptual article, Popova-Nowak and Cseh (2015) emphasized that there is a limited use of multidisciplinary perspectives to investigate and explain the processes and importance of utilizing the available capabilities and resources and of creating contexts where learning is “attractive to individual agents so that they can be more engaged in exploring ways in which they can contribute through their learning to the ongoing renewal of organizational routines and practices” (Antonacopoulou and Chiva 2007, p. 289).

Despite the importance of e-learning, the lack of systematic reviews in this area significantly hinders research on the highly promising value of e-learning capabilities for efficiently supporting organizational learning. This gap leaves practitioners and researchers in uncharted territories when faced with the task of implementing e-learning designs or deciding on their digital learning strategies to enhance the learning flow of their organizations. Hence, in order to derive meaningful theoretical and practical implications, as well as to identify important areas for future research, it is critical to understand how the core capabilities pertinent to e-learning possess the capacity to enhance organizational learning.

In this paper, we define e-learning enhanced organizational learning (eOL) as the utilization of digital technologies to enhance the process of improving actions through better knowledge and understanding in an organization. In recent years, a significant body of research has focused on the intersection of e-learning and organizational learning (e.g., Khandakar and Pangil 2019; Lin et al. 2019; Menolli et al. 2020; Turi et al. 2019; Xiang et al. 2020). However, there is a lack of systematic work that summarizes and conceptualizes the results in order to support organizations that want to move from being information-based enterprises to being knowledge-based ones (El Kadiri et al. 2016). In particular, recent technological advances have led to an increase in research that leverages e-learning capacities to support organizational learning, from virtual reality (VR) environments (Costello and McNaughton 2018; Muller Queiroz et al. 2018) to mobile computing applications (Renner et al. 2020) to adaptive learning and learning analytics (Zhang et al. 2019). These studies support different skills, consider different industries and organizations, and utilize various capacities while focusing on various learning objectives (Garavan et al. 2019). Our literature review aims to tease apart these particularities and to investigate how these elements have been utilized over the past decade in eOL research. Therefore, in this review we aim to answer the following research questions (RQs):

- RQ1: What is the status of research at the intersection of e-learning and organizational learning, seen through the lens of areas of implementation (e.g., industries, public sector), technologies used, and methodologies (e.g., types of data and data analysis techniques employed)?
- RQ2: How can e-learning be leveraged to enhance the process of improving actions through better knowledge and understanding in an organization?

Our motivation for this work is based on the emerging developments in the area of learning technologies that have created momentum for their adoption by organizations. This paper provides a review of research on e-learning capabilities to enhance organizational learning with the purpose of summarizing the findings and guiding future studies. This study can provide a springboard for other scholars and practitioners, especially in the area of knowledge-based enterprises, to examine e-learning approaches by taking into consideration the prior and ongoing research efforts. Therefore, in this paper we present a systematic literature review (SLR) (Kitchenham and Charters 2007) on the confluence of e-learning and organizational learning that uncovers initial findings on the value of e-learning to support organizational learning while also delineating several promising research streams.

The rest of this paper is organized as follows. In the next section, we present the related background work. The third section describes the methodology used for the literature review and how the studies were selected and analyzed. The fourth section presents the research findings derived from the data analysis based on the specific areas of focus. In the fifth section, we discuss the findings, the implications for practice and research, and the limitations of the selected methodological approach. In the final section, we summarize the conclusions from the study and make suggestions for future work.

## Background and Related Work

### E-learning Systems

E-learning systems provide solutions that deliver knowledge and information, facilitate learning, and increase performance by developing appropriate knowledge flow inside organizations (Menolli et al. 2020). Putting into practice and appropriately managing technological solutions, processes, and resources are necessary for the efficient utilization of e-learning in an organization (Alharthi et al. 2019). Examples of e-learning systems that have been widely adopted by various organizations are Canvas, Blackboard, and Moodle. Such systems provide innovative services for students, employees, managers, instructors, institutions, and other actors to support and enhance the learning processes and facilitate efficient knowledge flow (Garavan et al. 2019). Functionalities, such as creating modules to organize mini course information and learning materials or communication channels such as chat, forums, and video exchange, allow instructors and managers to develop appropriate training and knowledge exchange (Wang et al. 2011). Nowadays, the utilization of various e-learning capabilities is a commodity for supporting organizational and workplace learning. Such learning refers to training or knowledge development (also known in the literature as learning and development, HR development, and corporate training: Smith and Sadler-Smith 2006; Garavan et al. 2019) that takes place in the context of work.

Previous studies have focused on evaluating e-learning systems that utilize various models and frameworks. In particular, the development of maturity models, such as the e-learning capability maturity model (eLCMM), addresses technology-oriented concerns (Hammad et al. 2017) by overcoming the limitations of the domain-specific models (e.g., game-based learning: Serrano et al. 2012) or more generic lenses such as the e-learning maturity model (Marshall 2006). The aforementioned models are very relevant since they focus on assessing the organizational capabilities for sustainably developing, deploying, and maintaining e-learning. In particular, the eLCMM focuses on assessing the maturity of adopting e-learning systems and adds a feedback building block for improving learners’ experiences (Hammad et al. 2017). Our proposed literature review builds on the previously discussed models, lenses, and empirical studies, and it provides a review of research on e-learning capabilities with the aim of enhancing organizational learning in order to complement the findings of the established models and guide future studies.

E-learning systems can be categorized into different types, depending on their functionalities and affordances. One very popular e-learning type is the learning management system (LMS), which includes a virtual classroom and collaboration capabilities and allows the instructor to design and orchestrate a course or a module. An LMS can be either proprietary (e.g., Blackboard) or open source (e.g., Moodle). These two types differ in their features, costs, and the services they provide; for example, proprietary systems prioritize assessment tools for instructors, whereas open-source systems focus more on community development and engagement tools (Alharthi et al. 2019). In addition to LMS, e-learning systems can be categorized based on who controls the pace of learning; for example, an institutional learning environment (ILE) is provided by the organization and is usually used for instructor-led courses, while a personal learning environment (PLE) is proposed by the organization and is managed personally (i.e., learner-led courses). Many e-learning systems use a hybrid version of ILE and PLE that allows organizations to have either instructor-led or self-paced courses.

Besides the controlled e-learning systems, organizations have been using environments such as social media (Qi and Chau 2016), massive open online courses (MOOCs) (Weinhardt and Sitzmann 2018) and other web-based environments (Wang et al. 2011) to reinforce their organizational learning potential. These systems have been utilized through different types of technology (e.g., desktop applications, mobile) that leverage the various capabilities offered (e.g., social learning, VR, collaborative systems, smart and intelligent support) to reinforce the learning and knowledge flow potential of the organization. Although there is a growing body of research on e-learning systems for organizational learning due to the increasingly significant role of skills and expertise development in organizations, the role and alignment of the capabilities of the various e-learning systems with the expected competency development remains underexplored.

### Organizational Learning

There is a large body of research on the utilization of technologies to improve the process and outcome dimensions of organizational learning (Crossan et al. 1999). Most studies have focused on the learning process and on the added value that new technologies can offer by replacing some of the face-to-face processes with virtual processes or by offering new, technology-mediated phases to the process (Menolli et al. 2020; Lau 2015) highlighted how VR capabilities can enhance organizational learning, describing the new challenges and frameworks needed in order to effectively utilize this potential. In the same vein, Zhang et al. (2017) described how VR influences reflective thinking and considered its indirect value to overall learning effectiveness. In general, contemporary research has investigated how novel technologies and approaches have been utilized to enhance organizational learning, and it has highlighted both the promises and the limitations of the use of different technologies within organizations.

In many organizations, alignment with the established infrastructure and routines, and adoption by employees are core elements for effective organizational learning (Wang et al. 2011). Strict policies, low digital competence, and operational challenges are some of the elements that hinder e-learning adoption by organizations (Garavan et al. 2019; Wang 2018) demonstrated the importance of organizational, managerial, and job support for utilizing individual and social learning in order to increase the adoption of organizational learning. Other studies have focused on the importance of communication through different social channels to develop understanding of new technology, to overcome the challenges employees face when engaging with new technology, and, thereby, to support organizational learning (Menolli et al. 2020). By considering the related work in the area of organizational learning, we identified a gap in aligning an organization’s learning needs with the capabilities offered by the various technologies. Thus, systematic work is needed to review e-learning capabilities and how these capabilities can efficiently support organizational learning.

### E-learning Systems to Enhance Organizational Learning

When considering the interplay between e-learning systems and organizational learning, we observed that a major challenge for today’s organizations is to switch from being information-based enterprises to become knowledge-based enterprises (El Kadiri et al. 2016). Unidirectional learning flows, such as formal and informal training, are important but not sufficient to cover the needs that enterprises face (Manuti et al. 2015). To maintain enterprises’ competitiveness, enterprise staff have to operate in highly intense information and knowledge-oriented environments. Traditional learning approaches fail to substantiate learning flow on the basis of daily evidence and experience. Thus, novel, ubiquitous, and flexible learning mechanisms are needed, placing humans (e.g., employees, managers, civil servants) at the center of the information and learning flow and bridging traditional learning with experiential, social, and smart learning.

Organizations consider lack of skills and competences as being the major knowledge-related factors hampering innovation (El Kadiri et al. 2016). Thus, solutions need to be implemented that support informal, day-to-day, and work training (e.g., social learning, collaborative learning, VR/AR solutions) in order to develop individual staff competences and to upgrade the competence affordances at the organizational level. E-learning-enhanced organizational learning has been delivered primarily in the form of web-based learning (El Kadiri et al. 2016). More recently, the TEL tools portfolio has rapidly expanded to make more efficient joint use of novel learning concepts, methodologies, and technological enablers to achieve more direct, effective, and lasting learning impacts. Virtual learning environments, mobile-learning solutions, and AR/VR technologies and head-mounted displays have been employed so that trainees are empowered to follow their own training pace, learning topics, and assessment tests that fit their needs (Costello and McNaughton 2018; Mueller et al. 2011; Muller Queiroz et al. 2018). The expanding use of social networking tools has also brought attention to the contribution of social and collaborative learning (Hester et al. 2016; Wei and Ram 2016).

**Fig. 1:**
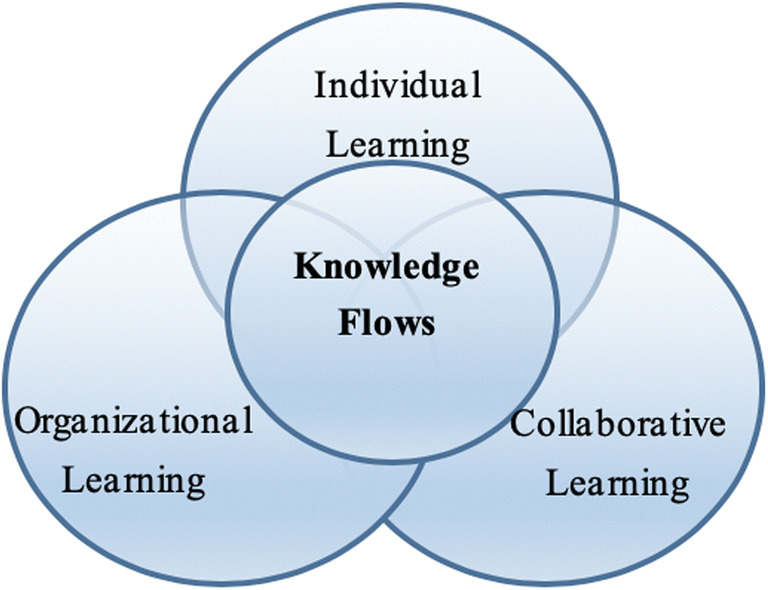
Representation of the combination of enterprise learning and knowledge flows. (adapted from Goggins et al. 2013)

Contemporary learning systems supporting adaptive, personalized, and collaborative learning expand the tools available in eOL and contribute to the adoption, efficiency, and general prospects of the introduction of TEL in organizations (Cheng et al. 2011). In recent years, eOL has emphasized how enterprises share knowledge internally and externally, with particular attention being paid to systems that leverage collaborative learning and social learning functionalities (Qi and Chau 2016; Wang 2011). This is the essence of computer-supported collaborative learning (CSCL). The CSCL literature has developed a framework that combines individual learning, organizational learning, and collaborative learning, facilitated by establishing adequate learning flows and emerges effective learning in an enterprise learning (Goggins et al. 2013), in Fig. 1.

Establishing efficient knowledge and learning flows is a primary target for future data-driven enterprises (El Kadiri et al. 2016). Given the involved knowledge, the human resources, and the skills required by enterprises, there is a clear need for continuous, flexible, and efficient learning. This can be met by contemporary learning systems and practices that provide high adoption, smooth usage, high satisfaction, and close alignment with the current practices of an enterprise. Because the required competences of an enterprise evolve, the development of competence models needs to be agile and to leverage state-of-the art technologies that align with the organization’s processes and models. Therefore, in this paper we provide a review of the eOL research in order to summarize the findings, identify the various capabilities of eOL, and guide the development of organizational learning in future enterprises as well as in future studies.

## Methodology

To answer our research questions, we conducted an SLR, which is a means of evaluating and interpreting all available research relevant to a particular research question, topic area, or phenomenon of interest. A SLR has the capacity to present a fair evaluation of a research topic by using a trustworthy, rigorous, and auditable methodology (Kitchenham and Charters 2007). The guidelines used (Kitchenham and Charters 2007) were derived from three existing guides adopted by medical researchers. Therefore, we adopted SLR guidelines that follow transparent and widely accepted procedures (especially in the area of software engineering and information systems, as well as in e-learning), minimize potential bias (researchers), and support reproducibility (Kitchenham and Charters 2007). Besides the minimization of bias and support for reproducibility, an SLR allows us to provide information about the impact of some phenomenon across a wide range of settings, contexts, and empirical methods. Another important advantage is that, if the selected studies give consistent results, SLRs can provide evidence that the phenomenon is robust and transferable (Kitchenham and Charters 2007).

### Article Collection

Several procedures were followed to ensure a high-quality review of the literature of eOL. A comprehensive search of peer-reviewed articles was conducted in February 2019 (short papers, posters, dissertations, and reports were excluded), based on a relatively inclusive range of key terms: “organizational learning” & “elearning”, “organizational learning” & “e-learning”, “organisational learning” & “elearning”, and “organisational learning” & “e-learning”. Publications were selected from 2010 onwards, because we identified significant advances since 2010 (e.g., MOOCs, learning analytics, personalized learning) in the area of learning technologies. A wide variety of databases were searched, including SpringerLink, Wiley, ACM Digital Library, IEEE Xplore, Science Direct, SAGE, ERIC, AIS eLibrary, and Taylor & Francis. The selected databases were aligned with the SLR guidelines (Kitchenham and Charters 2007) and covered the major venues in IS and educational technology (e.g., a basket of eight IS journals, the top 20 journals in the Google Scholar IS subdiscipline, and the top 20 journals in the Google Scholar Educational Technology subdiscipline). The search process uncovered 2,347 peer-reviewed articles.

### Inclusion and Exclusion Criteria

The selection phase determines the overall validity of the literature review, and thus it is important to define specific inclusion and exclusion criteria. As Dybå and Dingsøyr (2008) specified, the quality criteria should cover three main issues – namely, rigor, credibility, and relevance – that need to be considered when evaluating the quality of the selected studies. We applied eight quality criteria informed by the proposed Critical Appraisal Skills Programme (CASP) and related works (Dybå and Dingsøyr 2008). Table 1 presents these criteria.

**Table 1:** Quality criteria

1.	Does the study clearly address the research problem?
2.	Is there a clear statement of the aims of the research?
3.	Is there an adequate description of the context in which the research was carried out?
4.	Was the research design appropriate to address the aims of the research?
5.	Does the study clearly determine the research methods (subjects, instruments, data collection, data analysis)?
6.	Was the data analysis sufficiently rigorous?
7.	Is there a clear statement of findings?
8.	Is the study of value for research or practice?

**Fig. 2:**
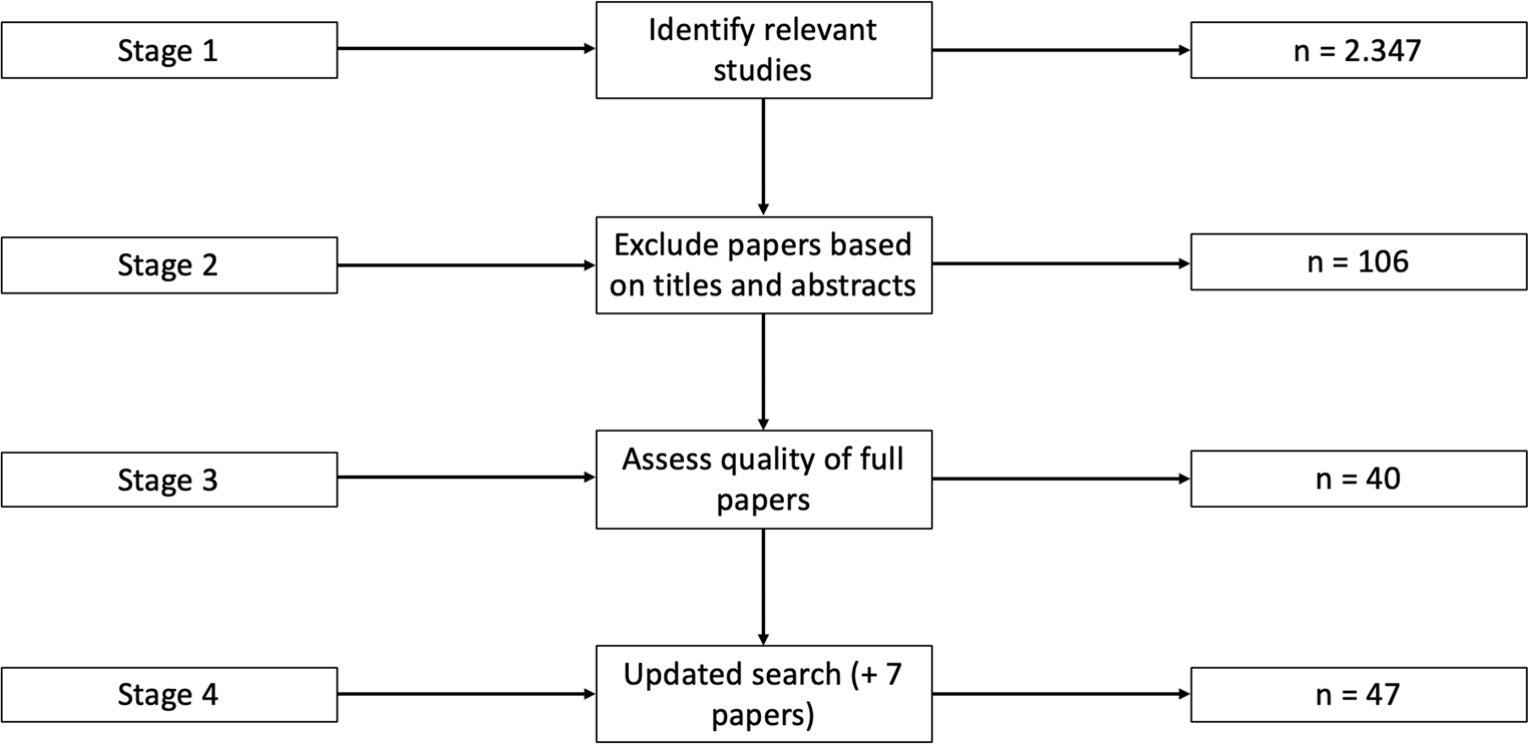
Stages of the selection process

Therefore, studies were eligible for inclusion if they were focused on eOL. The aforementioned criteria were applied in stages 2 and 3 of the selection process (see Fig. 2), when we assessed the papers based on their titles and abstracts, and read the full papers. From March 2020, we performed an additional search (stage 4) following the same process for papers published after the initial search period (i.e., 2010–February 2019). The additional search returned seven papers. Figure 2 summarizes the stages of the selection process.

### Analysis

Each collected study was analyzed based on the following elements: study design (e.g., experiment, case study), area (e.g., IT, healthcare), technology (e.g., wiki, social media), population (e.g., managers, employees), sample size, unit of analysis (individual, firm), data collections (e.g., surveys, interviews), research method, data analysis, and the main research objective of the study. It is important to highlight that the articles were coded based on the reported information, that different authors reported information at different levels of granularity (e.g., an online system vs. the name of the system), and that in some cases the information was missing from the paper. Overall, we endeavored to code the articles as accurately and completely as possible.

The coding process was iterative with regular consensus meetings between the two researchers involved. The primary coder prepared the initial coding for a number of articles and both coders reviewed and agreed on the coding in order to reach the final codes presented in the Appendix. Disagreements between the coders and inexplicit aspects of the reviewed papers were discussed and resolved in regular consensus meetings. Although this process did not provide reliability indices (e.g., Cohen’s kappa), it did provide certain reliability in terms of consistency of the coding and what Krippendorff (2018) stated as the reliability of “the degree to which members of a designated community concur on the readings, interpretations, responses to, or uses of given texts or data”, which is considered acceptable research practice (McDonald et al. 2019).

## Findings

In this section, we present the detailed results of the analysis of the 47 papers. Analysis of the studies was performed using non-statistical methods that considered the variables reported in the Appendix. This section is followed by an analysis and discussion of the categories.

### Sample Size and Population Involved

The categories related to the sample of the articles and included the number of participants in each study (size), their position (e.g., managers, employees), and the area/topic covered by the study. The majority of the studies involved employees (29), with few studies involving managers (6), civil servants (2), learning specialists (2), clients, and researchers. Regarding the sample size, approximately half of the studies (20) were conducted with fewer than 100 participants; some (12) can be considered large-scale studies (more than 300 participants); and only a few (9) can be considered small scale (fewer than 20 participants). In relation to the area/topic of the study, most studies (11) were conducted in the context of the IT industry, but there was also good coverage of other important areas (i.e., healthcare, telecommunications, business, public sector). Interestingly, several studies either did not define the area or were implemented in a generic context (sector-agnostic studies, n = 10), and some studies were implemented in a multi-sector context (e.g., participants from different sections or companies, n = 4).

### Research Methods

When assessing the status of research for an area, one of the most important aspects is the methodology used. By “method” in the Appendix, we refer to the distinction between quantitative, qualitative, and mixed methods research. In addition to the method, in our categorization protocol we also included “study design” to refer to the distinction between survey studies (i.e., those that gathered data by asking a group of participants), experiments (i.e., those that created situations to record beneficial data), and case studies (i.e., those that closely studied a group of individuals).

Based on this categorization, the Appendix shows that the majority of the papers were quantitative (34) and qualitative (7), with few studies (6) utilizing mixed methods. Regarding the study design, most of the studies were survey studies (26), 13 were case studies, and fewer were experiments (8). For most studies, the individual participant (40) was the unit of analysis, with few studies having the firm as the unit of analysis, and only one study using the training session as a unit of analysis. Regarding the measures used in the studies, most utilized surveys (39), with 11 using interviews, and only a few studies using field notes from focus groups (2) and log files from the systems (2). Only eight studies involved researchers using different measures to triangulate or extend their findings. Most articles used structural equation modeling (SEM) (17) to analyze their data, with 13 studies employing descriptive statistics, seven using content analysis, nine using regression analysis or analyses of variances/covariance, and one study using social network analysis (SNA).

### Technologies

Concerning the technology used, most of the studies (17) did not study a specific system, referring instead in their investigation to a generic e-learning or technological solution. Several studies (9) named web-based learning environments, without describing the functionalities of the identified system. Other studies focused on online learning environments (4), collaborative learning systems (3), social learning systems (3), smart learning systems (2), podcasting (2), with the rest of the studies using a specific system (e.g., a wiki, mobile learning, e-portfolios, Second Life, web application).

### Research Objectives

The research objectives of the studies could be separated into six main categories. The first category focuses on the intention of the employees to use the technology (9); the second focuses on the performance of the employees (8); the third focuses on the value/outcome for the organization (4); the fourth focuses on the actual usage of the system (7); the fifth focuses on employees’ satisfaction (4); and the sixth focuses on the ability of the proposed system to foster learning (9). In addition to these six categories, we also identified studies that focused on potential barriers for eOL in organizations (Stoffregen et al. 2016), the various benefits associated with the successful implementation of eOL (Liu et al. 2012), the feasibility of eOL (Kim et al. 2014; Mueller et al. 2011), and the alignment of the proposed innovation with the other processes and systems in the organization (Costello and McNaughton 2018).

### E-learning Capabilities in Various Organizations and for Various Objectives

The technology used has an inherent role for both the organization and the expected eOL objective. E-learning systems are categorized based on their functionalities and affordances. Based on the information reported in the selected papers, we ranked them based on the different technologies and functionalities (e.g., collaborative, online, smart). To do so, we focused on the main elements described in the selected paper; for instance, a paper that described the system as wiki-based or indicated that the system was Second Life was ranked as such, rather than being added to collaborative systems or social learning respectively. We did this because we wanted to capture all the available information since it gave us additional insights (e.g., Second Life is both a social and a VR system).

**Fig. 3:**
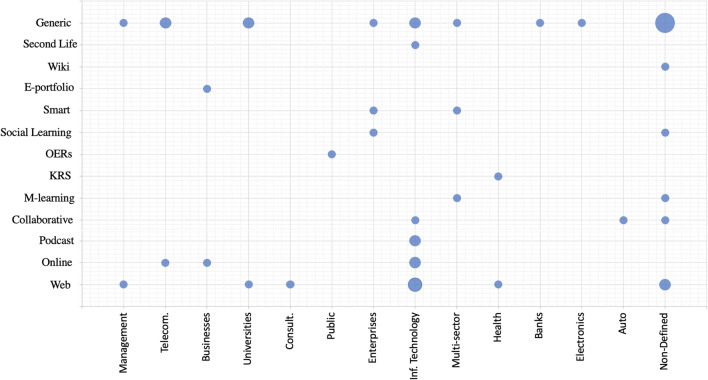
Association of the different e-learning technologies with the industries to which they are applied in the various studies. Note: The size of the circles depicts the frequency of studies, with the smallest circle representing one study and the largest representing six studies. The mapping is extracted from the data in the Appendix, which outlines the papers that belong in each of the circles

To investigate the connection between the various technologies used to enhance organizational learning and their application in the various organizations, we utilized the coding (see Appendix) and mapped the various e-learning technologies (or their affordances) with the research industries to which they applied (Fig. 3). There was occasionally a lack of detailed information about the capabilities of the e-learning systems applied (e.g., generic, or a web application, or an online system), which limited the insights. Figure 3 provides a useful mapping of the confluence of e-learning technologies and their application in the various industries.

**Fig. 4:**
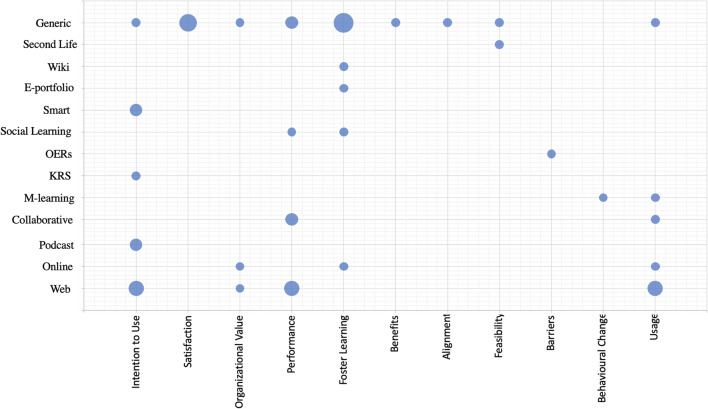
Association of the different e-learning technologies with the objectives investigated in the various studies. Note: The size of the circles depicts the frequency of studies, with the smallest circle representing one study and the largest representing five studies. The mapping is extracted from the data in the Appendix, which outlines the papers that belong in each of the circles

To investigate the connection between the various technologies used to enhance organizational learning and their intended objectives, we utilized the coding of the articles (see Appendix) and mapped the various e-learning technologies (or their affordances) with the intended objectives, as reported in the various studies (Fig. 4). The results in Fig. 4 show the objectives that are central in eOL research (e.g., performance, fostering learning, adoption, and usage) as well as those objectives on which few studies have focused (e.g., alignment, feasibility, behavioral change). In addition, the results also indicate the limited utilization of the various e-learning capabilities (e.g., social, collaborative, smart) to achieve objectives connected with those capabilities (e.g., social learning and behavioral change, collaborative learning, and barriers).

## 5. Discussion

After reviewing the 47 identified articles in the area of eOL, we can observe that all the works acknowledge the importance of the affordances offered by different e-learning technologies (e.g., remote collaboration, anytime anywhere), the importance of the relationship between eOL and employees’ satisfaction and performance, and the benefits associated with organizational value and outcome. Most of the studies agree that eOL provides employees, managers, and even clients with opportunities to learn in a more differentiated manner, compared to formal and face-to-face learning. However, how the organization adopts and puts into practice these capabilities to leverage them and achieve its goals are complex and challenging procedures that seem to be underexplored.

Several studies (Lee et al. 2015a; Muller Queiroz et al. 2018; Tsai et al. 2010) focused on the positive effect of perceived managerial support, perceived usefulness, perceived ease of use, and other technology acceptance model (TAM) constructs of the e-learning system in supporting all three levels of learning (i.e., individual, collaborative, and organizational). Another interesting dimension highlighted by many studies (Choi and Ko 2012; Khalili et al. 2012; Yanson and Johnson 2016) is the role of socialization in the adoption and usage of the e-learning systems that offer these capabilities. Building connections and creating a shared learning space in the e-learning system is challenging but also critical for the learners (Yanson and Johnson 2016). This is consistent with the expectancy-theoretical explanation of how social context impacts on employees’ motivation to participate in learning (Lee et al. 2015a; Muller Queiroz et al. 2018).

The organizational learning literature suggests that e-learning may be more appropriate for the acquisition of certain types of knowledge than others (e.g., procedural vs. declarative, or hard-skills vs. soft-skills); however, there is no empirical evidence for this (Yanson and Johnson 2016). To advance eOL research, there is a need for a significant move to address complex, strategic skills by including learning and development professionals (Garavan et al. 2019) and by developing strategic relationships. Another important element is to utilize e-learning technology that addresses and integrates organizational, individual, and social perspectives in eOL (Wang 2011). This is also identified in our literature review since we found only limited specialized e-learning systems in domain areas that have traditionally benefited from such technology. For instance, although there were studies that utilized VR environments (Costello and McNaughton 2018; Muller Queiroz et al. 2018) and video-based learning systems (Wei et al. 2013; Wei and Ram 2016), there was limited focus in contemporary eOL research on how specific affordances of the various environments that are used in organizations (e.g., Carnetsoft, Outotec HSC, and Simscale for simulations of working environments; or Raptivity, YouTube, and FStoppers to gain specific skills and how-to knowledge) can benefit the intended goals or be integrated with the unique qualities of the organization (e.g., IT, healthcare).

For the design and the development of the eOL approach, the organization needs to consider the alignment of individual learning needs, organizational objectives, and the necessary resources (Wang 2011). To achieve this, it is advisable for organizations to define the expected objectives, catalogue the individual needs, and select technologies that have the capacity to support and enrich learners with self-directed and socially constructed learning practices in the organization (Wang 2011). This needs to be done by taking into consideration that on-demand eOL is gradually replacing the classic static eOL curricula and processes (Dignen and Burmeister 2020).

Another important dimension of eOL research is the lenses used to approach effectiveness. The selected papers approached effectiveness with various objectives, such as fostering learning, usage of the e-learning system, employees’ performance, and the added organizational value (see Appendix). To measure these indices, various metrics (quantitative, qualitative, and mixed) have been applied. The qualitative dimensions emphasize employees’ satisfaction and system usage (e.g., Menolli et al. 2020; Turi et al. 2019), as well as managers’ perceived gained value and benefits (e.g., Lee et al. 2015b; Xiang et al. 2020) and firms’ perceived effective utilization of eOL resources (López-Nicolás and Meroño-Cerdán 2011). The quantitative dimensions focus on usage, feasibility, and experience at different levels within an organization, based on interviews, focus groups, and observations (Costello and McNaughton 2018; Michalski 2014; Stoffregen et al. 2016). However, it is not always clear the how eOL effectiveness has been measured, nor the extent to which eOL is well aligned with and is strategically impactful on delivering the strategic agenda of the organization (Garavan et al. 2019).

Research on digital technologies is developing rapidly, and big data and business analytics have the potential to pave the way for organizations’ digital transformation and sustainable development (Mikalef et al. 2018; Pappas et al. 2018); however, our review finds surprisingly limited use of big data and analytics in eOL. Despite contemporary e-learning systems adopting data-driven mechanisms, as well as advances in learning analytics (Siemens and Long 2011), the results of our analysis indicate that learner-generated data in the context of eOL are used in only a few studies to extract very limited insights with respect to the effectiveness of eOL and the intended objectives of the respective study (Hung et al. 2015; Renner et al. 2020; Rober and Cooper 2011). Therefore, eOL research needs to focus on data-driven qualities that will allow future researchers to gain deeper insights into which capabilities need to be developed to monitor the effectiveness of the various practices and technologies, their alignment with other functions of the organization, and how eOL can be a strategic and impactful vehicle for materializing the strategic agenda of the organization.

### Status of eOL Research

The current review suggests that, while the efficient implementation of eOL entails certain challenges, there is also a great potential for improving employees’ performance as well as overall organizational outcome and value. There are also opportunities for improving organizations’ learning flow, which might not be feasible with formal learning and training. In order to construct the main research dimensions of eOL research and to look more deeply at the research objectives of the studies (the information we coded as objectives in the Appendix), we performed a content analysis and grouped the research objectives. This enabled us to summarize the contemporary research on eOL according to five major categories, each of which is describes further below. As the research objectives of the published work shows, the research on eOL conducted during the last decade has particularly focused on the following five directions.

1. Investigating the capabilities of different technologies in different organizations.
  Research has particularly focused on how easy the technology is to use, on how useful it is, or on how well aligned/integrated it is with other systems and processes within the organization. In addition, studies have used different learning technologies (e.g., smart, social, personalized) to enhance organizational learning in different contexts and according to different needs. However, most works have focused on affordances such as remote training and the development of static courses or modules to share information with learners. Although a few studies have utilized contemporary e-learning systems (see Appendix), even in these studies there is a lack of alignment between the capabilities of those systems (e.g., open online course, adaptive support, social and collaborative learning) and the objectives and strategy of the organization (e.g., organizational value, fostering learning).
2. Enriching the learning flow and learning potential in different levels within an organization.
  The reviewed work has emphasized how different factors contribute to different levels of organizational learning, and it has focused on practices that address individual, collaborative, and organizational learning within the structure of the organization. In particular, most of the reviewed studies recognize that organizational learning occurs at multiple levels: individual, team (or group), and organization. In other words, although each of the studies carried out an investigation within a given level (except for Garavan et al. 2019), there is a recognition and discussion of the different levels. Therefore, the results align with the 4I framework of organizational learning that recognizes how learning across the different levels is linked by social and psychological processes: intuiting, interpreting, integrating, and institutionalizing (the 4Is) (Crossan et al. 1999). However, most of the studies focused on the institutionalizing-intuiting link (i.e., top-down feedback); moreover, no studies focused on contemporary learning technologies and processes that strengthen the learning flow (e.g., self-regulated learning).
3. Identifying critical aspects for effective eOL.
  There is a considerable amount of predominantly qualitative studies that focus on potential barriers to eOL implementation as well as on the risks and requirements associated with the feasibility and successful implementation of eOL. In the same vein, research has emphasized the importance of alignment of eOL (both in processes and in technologies) within the organization. These critical aspects for effective eOL are sometimes the main objectives of the studies (see Appendix). However, most of the elements relating to the effectiveness of eOL were measured with questionnaires and interviews with employees and managers, and very little work was conducted on how to leverage the digital technologies employed in eOL, big data, and analytics in order to monitor the effectiveness of eOL.
4. Implementing employee-centric eOL.
  In most of the studies, the main objective was to increase employees’ adoption, satisfaction, and usage of the e-learning system. In addition, several studies focused on the e-learning system’s ability to improve employees’ performance, increase the knowledge flow in the organization, and foster learning. Most of the approaches were employee-centric, with a small amount of studies focusing on managers and the firm in general. However, employees were seen as static entities within the organization, with limited work investigating how eOL-based training exposes employees to new knowledge, broadens their skills repertoire, and has tremendous potential for fostering innovation (Lin and Sanders 2017).
5. Achieving goals associated with the value creation of the organization.
  A considerable number of studies utilized the firm (rather than the individual employee) as the unit of analysis. Such studies focused on how the implementation of eOL can increase employee performance, organizational value, and customer value. Although this is extremely helpful in furthering knowledge about eOL technologies and practices, a more granular investigation of the different e-learning systems and processes to address the various goals and strategies of the organization would enable researchers to extract practical insights on the design and implementation of eOL.

### Research Agenda

**Fig. 5:**
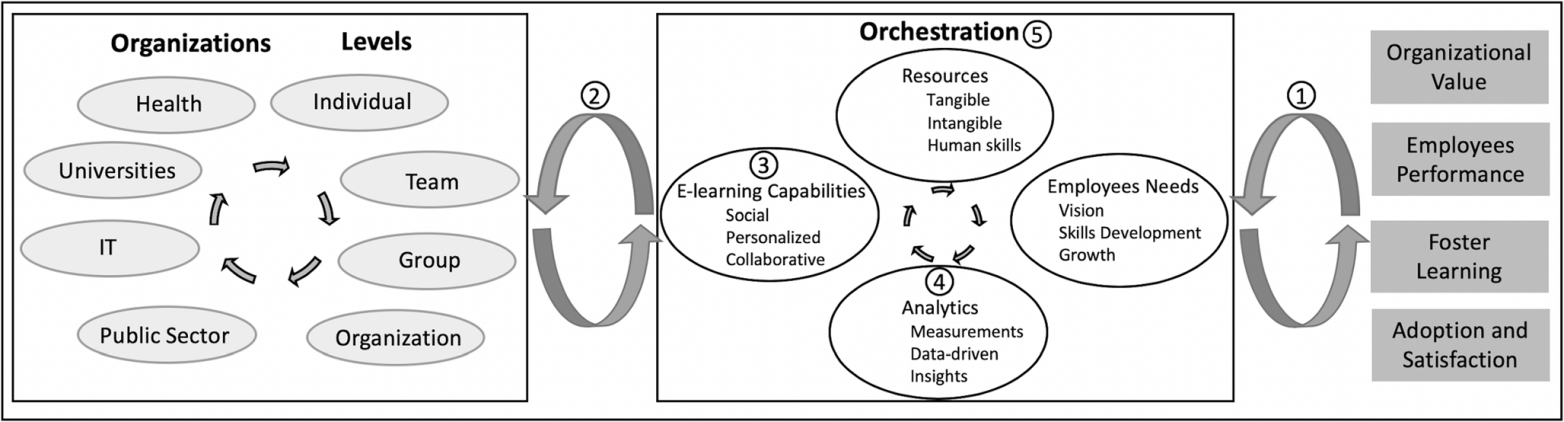
E-learning capabilities to enhance organizational research agenda

By conducting an SLR and documenting the eOL research of the last decade, we have identified promising themes of research that have the potential to further eOL research and practice. To do so, we define a research agenda consisting of five thematic areas of research, as depicted in the research framework in Fig. 5, and we provide some suggestions on how researchers could approach these challenges. In this visualization of the framework, on the left side we present the organizations as they were identified from our review (i.e., area/topic category in the Appendix) and the multiple levels where organizational learning occurs (Costello and McNaughton 2018). On the right side, we summarize the objectives as they were identified from our review (i.e., the objectives category in the Appendix). In the middle, we depict the orchestration that was conducted and how potential future research on eOL can improve the orchestration of the various elements and accelerate the achievement of the intended objectives. In particular, our proposed research agenda includes five research themes discussed in the following subsections.

#### Theme 1: Couple E-learning Capabilities With the Intended Goals

The majority of the eOL studies either investigated a generic e-learning system using the umbrella term “e-learning” or did not provide enough details about the functionalities of the system (in most cases, it was simply defined as an online or web system). This indicates the very limited focus of the eOL research on the various capabilities of e-learning systems. In other words, the literature has been very detailed on the organizational value and employees’ acceptance of the technology, but less detailed on the capabilities of this technology that needs to be put into place to achieve the intended goals and strategic agenda. However, the capabilities of the e-learning systems and their use are not one-size-fits-all, and the intended goals (to obtain certain skills and competences) and employees’ needs and backgrounds play a determining role in the selection of the e-learning system (Al-Fraihat et al. 2020).

Only in a very few studies (Mueller et al. 2011; Renner et al. 2020) were the capabilities of the e-learning solutions (e.g., mobile learning, VR) utilized, and the results were found to significantly contribute to the intended goals. The intended knowledge can be procedural, declarative, general competence (e.g., presentation, communication, or leadership skills) or else, and its particularities and the pedagogical needs of the intended knowledge (e.g., a need for summative/formative feedback or for social learning support) should guide the selection of the e-learning system and the respective capabilities. Therefore, future research needs to investigate how the various capabilities offered by contemporary learning systems (e.g., assessment mechanisms, social learning, collaborative learning, personalized learning) can be utilized to adequately reinforce the intended goals (e.g., to train personnel to use a new tool, to improve presentation skills).

#### Theme 2: Embrace the Particularities of the Various Industries

Organizational learning entails sharing knowledge and enabling opportunities for growth at the individual, group, team, and organizational levels. Contemporary e-learning systems provide the medium to substantiate the necessary knowledge flow within organizations and to support employees’ overall learning. From the selected studies, we can infer that eOL research is either conducted in an industry-agnostic context (either generic or it was not properly reported) or there is a focus on the IT industry (see Appendix). However, when looking at the few studies that provide results from different industries (Garavan et al. 2019; Lee et al. 2014), companies indicate that there are different practices, processes, and expectations, and that employees have different needs and perceptions with regards to e-learning systems and eOL in general. Such particularities influence the perceived dimensions of a learning organization. Some industries noted that eOL promoted the development of their learning organizations, whereas others reported that eOL did not seem to contribute to their development as a learning organization (Yoo and Huang 2016). Therefore, it is important that the implementation of organizational learning embraces the particularities of the various industries and future research needs to identify how the industry-specific characteristics can inform the design and development of organizational learning in promoting an organization’s goals and agenda.

#### Theme 3: Utilize E-learning Capabilities to Implement Employee-centric Approaches

For efficient organizational learning to be implemented, the processes and technologies need to recognize that learning is linked by social and psychological processes (Crossan et al. 1999). This allows employees to develop learning in various forms (e.g., social, emotional, personalized) and to develop elements such as self-awareness, self-control, and interpersonal skills that are vital for the organization. Looking at the contemporary eOL research, we notice that the exploration of e-learning capabilities to nurture the aforementioned elements and support employee-centric approaches is very limited (e.g., personalized technologies, adaptive assessment). Therefore, future research needs to collect data to understand how e-learning capabilities can be utilized in relation to employees’ needs and perceptions in order to provide solutions (e.g., collaborative, social, adaptive) that are employee-centric and focused on development, and that have the potential to move away from standard one-size-fits-all e-learning solutions to personalized and customized systems and processes.

#### Theme 4: Employ Analytics-enabled eOL

There is a lot of emphasis on measuring, via various qualitative and quantitative metrics, the effectiveness of eOL implemented at different levels in organizations. However, most of these metrics come from surveys and interviews that capture employees’ and managers’ perceptions of various aspects of eOL (e.g., fostering of learning, organizational value, employees’ performance), and very few studies utilize analytics (Hung et al. 2015; Renner et al. 2020; Rober and Cooper 2011). Given how digital technologies, big data, and business analytics pave the way towards organizations’ digital transformation and sustainable development (Mikalef et al. 2018; Pappas et al. 2018), and considering the learning analytics affordances of contemporary e-learning systems (Siemens and Long 2011), future work needs to investigate how learner/employee-generated data can be employed to inform practice and devise more accurate and temporal effectiveness metrics when measuring the importance and impact of eOL.

#### Theme 5: Orchestrate the Employees’ Needs, Resources, and Objectives in eOL Implementation

While considerable effort has been directed towards the various building blocks of eOL implementation, such as resources (intangible, tangible, and human skills) and employees’ needs (e.g., vision, growth, skills development), little is known so far about the processes and structures necessary for orchestrating those elements in order to achieve an organization’s intended goals and to materialize its overall agenda. In other words, eOL research has been very detailed on some of the elements that constitute efficient eOL, but less so on the interplay of those elements and how they need to be put into place. Prior literature on strategic resource planning has shown that competence in orchestrating such elements is a prerequisite to successfully increasing business value (Wang et al. 2012). Therefore, future research should not only investigate each of these elements in silos, but also consider their interplay, since it is likely that organizations with similar resources will exert highly varied levels in each of these elements (e.g., analytics-enabled, e-learning capabilities) to successfully materialize their goals (e.g., increase value, improve the competence base of their employees, modernize their organization).

### Implications

Several implications for eOL have been revealed in this literature review. First, most studies agree that employees’ or trainees’ experience is extremely important for the successful implementation of eOL. Thus, keeping them in the design and implementation cycle of eOL will increase eOL adoption and satisfaction as well as reduce the risks and barriers. Another important implication addressed by some studies relates to the capabilities of the e-learning technologies, with easy-to-use, useful, and social technologies resulting in more efficient eOL (e.g., higher adoption and performance). Thus, it is important for organizations to incorporate these functionalities in the platform and reinforce them with appropriate content and support. This should not only benefit learning outcomes, but also provide the networking opportunities for employees to broaden their personal networks, which are often lost when companies move from face-to-face formal training to e-learning-enabled organizational learning.

### Limitations

This review has some limitations. First, we had to make some methodological decisions (e.g., selection of databases, the search query) that might lead to certain biases in the results. However, tried to avoid such biases by considering all the major databases and following the steps indicated by Kitchenham and Charters (2007). Second, the selection of empirical studies and coding of the papers might pose another possible bias. However, the focus was clearly on the empirical evidence, the terminology employed (“e-learning”) is an umbrella term that covers the majority of the work in the area, and the coding of papers was checked by two researchers. Third, some elements of the papers were not described accurately, leading to some missing information in the coding of the papers. However, the amount of missing information was very small and could not affect the results significantly. Finally, we acknowledge that the selected methodology (Kitchenham and Charters 2007) includes potential biases (e.g., false negatives and false positives), and that different, equally valid methods (e.g., Okoli and Schabram 2010) might have been used and have resulted in slightly different outcomes. Nevertheless, despite the limitations of the selected methodology, it is a well-accepted and widely used literature review method in both software engineering and information systems (Boell and Cecez-Kecmanovic 2014), providing certain assurance of the results.

## Conclusions and Future Work

We have presented an SLR of 47 contributions in the field of eOL over the last decade. With respect to RQ1, we analyzed the papers from different perspectives, such as research methodology, technology, industries, employees, and intended outcomes in terms of organizational value, employees’ performance, usage, and behavioral change. The detailed landscape is depicted in the Appendix and Figs. 3 and 4; with the results indicating the limited utilization of the various e-learning capabilities (e.g., social, collaborative) to achieve objectives connected with those capabilities (e.g., social learning and behavioral change, collaborative learning and overcoming barriers).

With respect to RQ2, we categorized the main findings of the selected papers into five areas that reflect the status of eOL research, and we have discussed the challenges and opportunities emerging from the current review. In addition, we have synthesized the extracted challenges and opportunities and proposed a research agenda consisting of five elements that provide suggestions on how researchers could approach these challenges and exploit the opportunities. Such an agenda will strengthen how e-learning can be leveraged to enhance the process of improving actions through better knowledge and understanding in an organization.

A number of suggestions for further research have emerged from reviewing prior and ongoing work on eOL. One recommendation for future researchers is to clearly describe the eOL approach by providing detailed information about the technologies and materials used, as well as the organizations. This will allow meta-analyses to be conducted and it will also identify the potential effects of a firm’s size or area on the performance and other aspects relating to organizational value. Future work should also focus on collecting and triangulating different types of data from different sources (e.g., systems’ logs). The reviewed studies were conducted mainly by using survey data, and they made limited use of data coming from the platforms; thus, the interpretations and triangulation between the different types of collected data were limited.

## Appendix

**Table:**

Study	Study Design	Area/ Topic	Technology	Population	Sample	Unit of Analysis	Data Coll. Tool	Method Type	Analysis	Objectives
Cheng et al. 2012	Survey	ND	Web	Mixed	222	Individ.	Surv.	Quant.	SEM	ItU
Mitić et al. 2017	Survey	MGM	Generic	Mg	380	Individ.	Surv.	Quant.	Reg.	Sat.
Navimipour and Zareie 2015	Survey	Telec.	Generic	Empl.	128	Individ.	Surv.	Quant.	SEM	Sat.
Yanson and Johnson 2016	Exp.	Bsn	Online	Stud.	143	Individ.	Surv.	Quant.	A-VA	Flearn
Iris and Vikas 2011	Survey	IT	Online	Empl.	500	Individ.	Surv.	Quant.	Reg.	OV
Alsabawy et al. 2013	Survey	Univ.	Web	Stud.	832	Individ.	Surv.	Quant.	SEM	OV
Jia et al. 2011	Exp.	IT	Web	Empl.	24	Individ.	Surv.	Quant.	Descr.	Per.
Wang et al. 2011	Exp.	IT	Web	Empl.	24	Individ.	Surv./Int.	Mixed	Descr.	Per.
Wei and Ram 2016	Exp.	IT	Podcast	Empl.	26	Individ.	Surv./Int.	Mixed	CA	ItU
Bologa and Lupu 2014	Casest	Cons.	Social	Empl.	12	Session	Int.	Qual.	Descr.	Per.
Cheng et al. 2011	Survey	Cons.	Web	Clients	222	Individ.	Surv.	Quant.	SEM	ItU
Lee et al. 2015b	Survey	ND	M-learn	Mg	342	Individ.	Surv.	Quant	Descr.	Usage
Choi and Ko 2012	Survey	ND	Collabor	ND	130	Individ.	Surv.	Quant.	SEM	Per.
Tsai et al. 2010	Survey	Health	KRS	Empl.	800	Individ.	Surv.	Quant.	SEM	ItU
Stoffregen et al. 2016	Casest	Public	Oers	Civil	68	Individ.	Int./FG	Qual.	CA	Barr.
López-Nicolás and Meroño-Cerdán 2011	Survey	Ent.	Generic	Empl.	317	Firm	Surv.	Quant.	SEM	Per.
Wang 2011	Exp.	ND	Social	Empl.	28	Individ.	Surv.	Quant.	Descr.	Per.
Lee et al. 2015a	Survey	Ent.	Smart	Mg	120	Individ.	Surv.	Quant.	SEM	ItU
Wang and Wang 2012	Exp.	Bsn	E-port.	Stud.	7	Individ.	Surv.	Quant.	Descr.	Flearn
Siadaty et al. 2010	Casest	Autom	Collabor.	Empl.	3	Firm	Int/FG	Qual.	Descr.	Usage
Subramaniam and Nakkeeran 2019	Casest	IT	Collabor.	Empl.	202	Individ.	Surv.	Quant.	Reg.	Per.
Kaschig et al. 2010	Casest	ND	Generic	Empl.	126	Individ.	Int.	Mixed	CA	Flearn
Khalili et al. 2012	Casest	ND	Wiki	Res.	16	Individ.	Surv.	Quant.	Descr.	Flearn
Qi and Chau 2016	Survey	Ent.	Social	Empl.	97	Individ.	Surv.	Quant.	SEM	Flearn
Wei et al. 2013	Survey	IT	Podcast	Empl.	12	Individ.	Int.	Qual.	CA	ItU
Costello & McNaughton 2018	Survey	ND	Generic	Learn	12	Firm	Int.	Qual.	CA	Align.
Mueller et al. 2011	Casest	IT	SL	ND	16	Individ.	Int.	Qual.	CA	Feas.
Hung et al. 2015	Casest	MGM	Web	ND	22	Individ.	Surv., log	Quant	Descr.	Usage
Lee et al. 2014	Survey	Multi-sector	Smart	Empl.	342	Individ.	Surv.	Quant.	SEM	ItU
Hester et al. 2016	Survey	ND	Generic	Learn	83	Individ.	Surv.	Quant.	SEM	ItU
Gal and Nachmias 2011	Survey	Telec.	Online	Empl.	294	Individ.	Surv.	Quant.	A-VA	Usage
Kim et al. 2014	Survey	IT	Generic	Empl.	550	Individ.	Surv.	Quant.	Descr.	Feas.
Rober and Cooper 2011	Casest	Health	Web	Empl.	40	Individ.	Log	Quant.	Descr.	Usage
Michalski 2014	Casest	ND	Generic	Empl.	15	Individ.	Int.	Qual.	CA	Usage
Joo et al. 2011	Survey	Electr.	Generic	Empl.	379	Individ.	Surv.	Quant.	SEM	Flearn
Liu et al. 2012	Survey	ND	Generic	Empl.	120	Firm	Surv.	Quant.	SEM	Benef.
Wang et al. 2010	Exp	IT	Web	Empl	24	Individ.	Surv., int.	Mixed	Descr.	Per.
Lin et al. 2019	Survey	Telec.	Generic	ND	297	Individ.	Surv.	Quant.	SEM	Sat.
Lai 2017	Survey	ND	Web	Civil	439	Individ.	Surv.	Quant.	SEM	ItU
Škerlavaj et al. 2010	Casest	IT	Generic	Empl.	93	Individ.	Surv.	Quant.	SNA	Flearn
Garavan et al. 2019	Casest	Multi-sector	Generic	Mgr, empl., learn.	440, 125, 30	Individ.	Int, Surv, Delphi	Mixed	A-VA, Reg.	Flearn
Renner et al. 2020	Casest	Multi-sector	M-learn	Empl	311	Individ.	Int., surv., log	Mixed	A-VA	Beh.
Xiang et al. 2020	Survey	Multi-sector	Generic	Mg	282	Firm	Surv.	Quant.	Reg.	OV
Menolli et al. 2020	Exp.	IT	Online	Empl.	18	Individ.	Surv.	Quant.	Descr.	Usage
Turi et al. 2019	Survey	Univ.	Generic	Empl.	137	Individ.	Surv.	Quant.	Reg.	OV
Khandakar and Pangil 2019	Survey	Banks	Generic	Mg	364	Firm	Surv.	Quant.	SEM	Flearn
Lin et al. 2019	Survey	IT	Generic	Empl	297	Individ.	Surv.	Quant.	SEM	Sat.

Survey = survey study; Exp. = experiment; CaseSt = case study; ND = non-defined; MGM = management; Telec. = telecommunication; Bsn = business; Univ. = university; Cons. = consulting; Public = public sector; Ent. = enterprise; Web = Web-based; KRS = knowledge repository system; OERs = open educational resources; SL = Second Life, Mg, = managers; Empl = employees; Stud = students; Res. = researchers; Learn. = learning specialists; Individ. = individual; Surv. = surveys; Int. = interviews; FG = focus groups; Log = log files; Obs. = observations; Reg. = regression analysis; Descr. = descriptive statistics; A-VA = analysis of variances/covariance; CA = content analysis; ItU = intention to use; Sat. = satisfaction; OV = organizational value; Per. = performance; Flearn = foster learning; Benef. = benefits; Align. = alignment; Feas. = feasibility; Barr. = barriers; Beh. = behavioral change
